# Local delivery of gaseous signaling molecules for orthopedic disease therapy

**DOI:** 10.1186/s12951-023-01813-6

**Published:** 2023-02-21

**Authors:** Jiaxuan Sun, Wenzhi Wang, Xianli Hu, Xianzuo Zhang, Chen Zhu, Jinming Hu, Ruixiang Ma

**Affiliations:** 1 Department of Orthopedics, The First Affiliated Hospital of University of Science and Technology of China (USTC), Division of Life Sciences and Medicine, University of Science and Technology of China, Hefei, 230001 Anhui China; 2 Department of Pharmacy, The First Affiliated Hospital of USTC, Division of Life Sciences and Medicine, CAS Key Laboratory of Soft Matter Chemistry, Department of Polymer Science and Engineering, University of Science and Technology of China, Hefei, 230001 Anhui China

**Keywords:** Gaseous signaling molecules, Orthopedic disorders, Drug delivery systems, Nitric oxide, Hydrogen sulfide, Carbon monoxide

## Abstract

Over the past decade, a proliferation of research has used nanoparticles to deliver gaseous signaling molecules for medical purposes. The discovery and revelation of the role of gaseous signaling molecules have been accompanied by nanoparticle therapies for their local delivery. While most of them have been applied in oncology, recent advances have demonstrated their considerable potential in diagnosing and treating orthopedic diseases. Three of the currently recognized gaseous signaling molecules, nitric oxide (NO), carbon monoxide (CO), and hydrogen sulfide (H_2_S), are highlighted in this review along with their distinctive biological functions and roles in orthopedic diseases. Moreover, this review summarizes the progress in therapeutic development over the past ten years with a deeper discussion of unresolved issues and potential clinical applications.

## Introduction

Since a fluid water environment inundates most cells, it is practical to transmit signals by hydrophilic molecules or ions to bind to the receptors on living membrane surfaces. However, the discovery of the endogenous production of nitric oxide (NO) and its subsequent physiological effects, such as blood-vessel relaxation [1], neurotransmitter release regulation [2], and macrophage activation [3], ignited our interest in gaseous signaling molecules considering that an adequately small and hydrophobic molecule can cross the plasma membrane and initiate intracellular cell modulation. Similarly, carbon monoxide (CO) and hydrogen sulfide (H_2_S), which are chiefly synthesized through enzymatic and nonenzymatic pathways, act as signaling molecules in the immune, nervous, and cardiovascular systems [4]. They do not bind to any specific receptors but only utilize simple and direct chemical reactions or posttranslation modifications to affect proteins and others. The three gaseous signaling molecules identified to date, NO, CO, and H_2_S, are not only involved in physiological processes but also overproduced in tumors [5] and many other pathological processes [6–9], such as NO in septic shock [10], suggesting a role in disease development. Additionally, other simple small molecular gases composed of common nonmetallic elements, such as methane, carbon dioxide, sulfur dioxide, and hydrogen, are also considered potential gaseous signaling molecules [11]. Their potential interaction with signaling pathways needs thorough investigation despite their endogenous nature of exhibiting physiological functions.

By supplying exogenous gaseous signaling molecules or scavenging endogenous ones, therapies relying on immunomodulation, neuromodulation and vascular modulation have recently presented considerable prospects. Since inhalation administration is the most direct and effective method concerning gaseous drugs, inhaled NO exceptionally relieves neonatal pulmonary hypertension [12]. With the ascent of the COVID-19 pandemic, a significant decrease in the available quantities of physiology NO and H_2_S in patients was observed [13]. Fortunately, inhaled NO supplementation has the potential to improve pathological states such as ischemia-hypoxia and inflammation in cardiovascular diseases and respiratory diseases [14]. Oral administration of nitroglycerin, a classical drug for the treatment of angina, can also exert therapeutic effects on gaseous signaling molecules. Nonetheless, this route is strictly restricted to systems with exposed mucous membranes due to the extremely short half-life of the diffusion-limited NO. Therefore, its high reactivity implies that gaseous signaling molecules should be released locally and controllably for efficient application to many other diseases. Notably, the burgeoning development of nanomaterials is hailed for inspiring this therapy to overcome the challenging problems of untargeted delivery, short half-life, and uncontrollable concentration. Hence, nanomaterials have far been excellent platforms for gas release [15].

The ability to move freely has frequently been synchronized as a crucial sign of splendid health. However, fractures, osteoarthritis (OA), rheumatoid arthritis (RA), osteoporosis (OSP), herniated discs, cervical spondylosis, and other musculoskeletal disorders are diminishing the quality of life today, with chief symptoms of pain and impaired movement [16]. A large variety of nanomaterials have been developed for the treatment of bone and connective tissue diseases [17], among which gaseous signaling molecule-releasing materials, especially NO-releasing nanoplatforms, have exhibited outstanding efficacy in inflammatory diseases and bone metabolic diseases [18]. Interestingly, the effects of NO vary at different concentrations [19], demonstrating antibacterial activity at unbearably higher concentrations than physiological concentrations, as do CO and H_2_S gases [20, 21]. Hence, gaseous signaling molecules releasing nanomaterials can also be adopted to wrestle with implant-associated infections or other infectious issues after surgical procedures while performing physiological modulation functions.

**Fig. 1:**
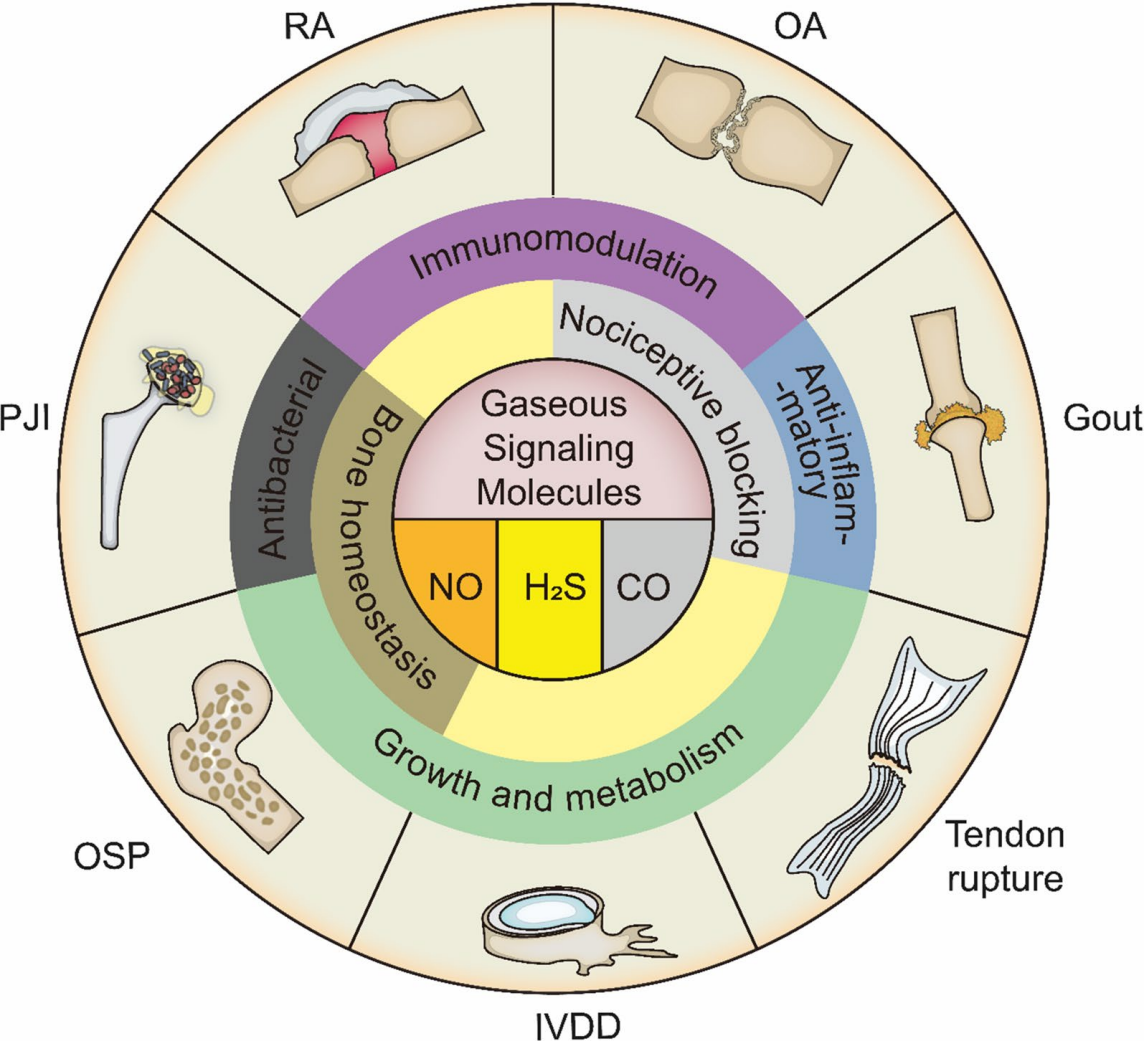
Applications of local delivery of gaseous signaling molecules in orthopedic diseases. *RA* rheumatoid arthritis, *OA* osteoarthritis, *IVDD* intervertebral disc degeneration, *OSP* osteoporosis, *PJI* periprosthetic joint infection, *NO* nitric oxide, *H*_*2*_*S* hydrogen sulfide, *CO* carbon monoxide

In this review, the vital roles of the three principal gaseous signaling molecules in orthopedic diseases are illuminated by analyzing the basic properties (Table 1) and principles of using gaseous signaling molecules in therapy and summarizing their novel decade-long existing therapeutic developments for orthopedic diseases (Fig. 1). Overall, this review aims to provide new insights into gas delivery strategies for bone, joint, and tendon diseases and the development of related drugs.

**Table 1:** Properties of gaseous signal molecules

Gas	NO	H_2_S	CO
Odor	Odorless	Rotten egg smell	Odorless
Toxicity: (Immediately dangerous to life or health values)	100 ppm	100 ppm	1200 ppm
Reactivity	Easily forms free radicals; reacts rapidly with oxygen and water	Dissociates HS^−^ with high reactivity; stability is affected by both pH and oxygen concentration	Relative biological inertness; Mostly forms coordination bonds with transition metal elements
Half-time in vivo	Intravascular: 2 ms [183] Extravascular: From 0.09 ms to > 2 ms [184]	Intravascular: 130.5–151.1 s [185] Extravascular: 2–10 min [186]	Intravascular: 300 min(COHb)[187] COHb Extravascular: unknow
Physiological concentration	100 pM-200 nM [189, 190]	50–160 μM (brain) [191]	0.5–1.5 μM [190]
Recommended practical therapeutic flux (measured in vitro)	Physiology: 0.05–1 μM/min Antibacterial: 1–10 μM/min	Physiology: 0.01–0.1 μM/min Antibacterial: ~ 0.8 μM/min	Physiology: 0.1–0.3 μM/min Antibacterial: ~ 20 μM/min

Toxicity data from the National Institute for Occupational Safety and Health (NIOSH)

## NO

### Generation of NO

**Fig. 2:**
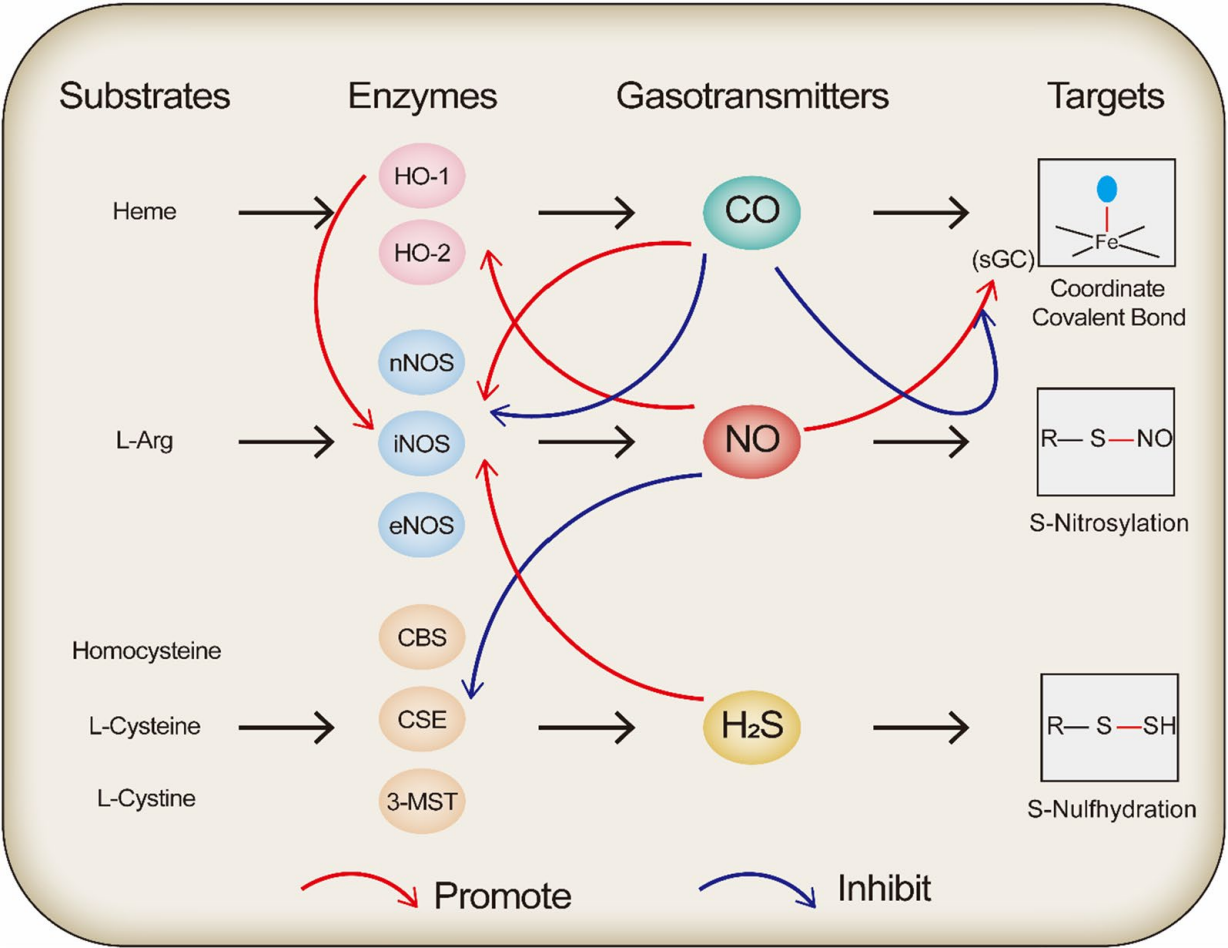
Main sources, signaling effects, and crosstalk of gaseous signal molecules. *HO-1* heme oxygenase-1, *HO-2* heme oxygenase-2, *nNOS* neuronal nitric oxide synthase, *iNOS* inducible nitric oxide synthase, *eNOS* endothelial nitric oxide synthase, *CBS* cystathionine-β-synthase, *CSE* cystathionine γ-lyase, *3-MST* mercaptopyruvate sulfurtransferase, *sGC* soluble guanylyl cyclase

The vast majority of NO in the body is produced by nitric oxide synthase (NOS), which comprises several isoforms of enzymes: endothelial NOS (eNOS), neuronal NOS (nNOS), inducible NOS (iNOS) (Fig. 2), and probably mitochondrial NOS (mtNOS) [22]. Cytochrome p450 within the enzyme acts as the center of redox reactions, converting L-arginine (L-Arg) substrate and oxygen to citrulline and NO in a two-step, five-electron oxidation reaction [23]. Under physiological conditions, eNOS and nNOS are consistently and stably expressed, and Ca^2+^-sensitivity enables the stimulated production of proportionally relevant low levels of NO. When cells encounter bacteria or inflammatory cytokines, iNOS expression is induced, and subsequently, high levels of NO are released.

### Physiological functions of NO

The earlier discovery of endothelium-derived relaxing factor (EDRF), a character later identified as NO [24], was a groundbreaking development in the field of gaseous signaling molecules. In the above process, acetylcholine released from neuronal nerve endings binds to receptors on endothelial cells to boost the output of NO from eNOS. Then, the produced NO diffuses into the smooth muscle cells and binds to iron at the active site of soluble guanylate cyclase (sGC), activating the generation of cGMP, which functions as a second messenger to regulate vascular smooth muscle tone. Additionally, the effect of NO is generally accomplished by the modification of proteins, mainly through nitrosylation of the sulfhydryl group of Cys residues [25] (Fig. 2).

### NO on immunity, inflammation, and bacteria

Regarding immune cells, NO is not simply a destroyer. High concentrations of NO are only immunosuppressive due to its cytotoxicity[26], while low concentrations of NO can selectively inhibit or induce the differentiation of T helper cells to some of the subtypes [27, 28]. NO is involved in the regulation of differentiation, proliferation, and immune activity of practically all immune cells, whether of the lymphoid or myeloid lineage [29]. Toll-like receptors in macrophages recognize the bacterial component and upregulate iNOS expression to produce more NO when bacterial infections occur. However, when bacteria-containing vesicles from intracellularly infected macrophages enter uninfected cells and form secondary vesicles, NO not only eases bacterial escape from the secondary vesicles but also hinders the fusion of phagolysosomes with secondary vesicles, both of which simplify cell-to-cell infection; thus, NO acts as a pathogen helper to accelerate the spread of infections [30]. In a study of inflammatory arthritis, the osteoclast precursor (OPC, BM CD11b^−/lo^ Ly6C^hi^) in bone marrow-derived cells, which has many osteoclast-specific receptors and can differentiate into osteoclasts in vivo, was identified. These osteoclast precursors from SKG mice with chronic spontaneous inflammatory arthritis inhibit the proliferation of CD4^+^ and CD8^+^ T cells by releasing NO[31], affirming the immunomodulatory role played by NO. The cells (almost all cells involved in immune processes) that produce NO and how their eNOS or iNOS are regulated have been revealed [32]. However, the role played by NO is unclear since immune cells are not only producers but also receivers of NO signals. The existence of multiple targets within the receiving cells and the diverse functions determined by precise concentrations of NO also hinder the understanding of the basic aspects of NO immunoregulation.

Inflammation is the immune system's primary combat mode of defending against pathogens or responding to tissue damage and is usually considered beneficial, although it sometimes causes inflammatory diseases. Shortly after the recognition of NO as a gaseous signaling molecule, its release in inflammatory tissues was discovered [33]. Henceforth, NO has been inextricably linked to inflammation and inflammatory diseases. Substantially, the expression of iNOS was discovered to be upregulated in tissues extricated from MRL-Ipr/lpr mouse models subject to multiple autoimmune diseases. In addition, the macrophages extracted from their peritoneum contained more active iNOS, and the cells had an enhanced NO release capacity. When NOS inhibitors were administered, the mice were free from glomerulonephritis, and inflammatory arthritis was also significantly attenuated [34]. Low concentrations of NO can suppress the expression of tissue inhibitor of metalloproteinase (TIMP)-1 and boost the expression and activity of matrix metalloproteinase (MMP)-9 and MMP-3 [35], contributing to strengthening extracellular matrix degradation and wound healing. However, when NO concentration is high enough to match the concentration during macrophage activation, MMPs are inactivated [36]. NO can also modify complex III of the mitochondrial respiratory complexes in chondrocytes, causing inhibition of its activity and alteration of the mitochondrial membrane potential, resulting in the inhibition of mitochondrial respiration and then chondrocyte apoptosis [37]. NO is associated with an increasing number of inflammatory signaling pathways. By regulating transcription factors, including NF-κB, AP-1, and Jak-STAT [38], NO exerts a significant influence on the inflammatory process and hence relays an enhanced certainty of the relevance of NO as an essential mediator and regulator of inflammation. Nevertheless, just as in the immune system, NO expresses antagonistic proinflammatory and anti-inflammatory properties at times [39]. Therefore, the effects of NO on specific inflammatory diseases require detailed examination.

NO and its derivatives perform antimicrobial activities through diverse targets. In addition to damaging bacterial cell membranes and cell walls during their entry into the bacteria via porins [40], they also intracellularly mediate the release and depletion of Fe^2+^ by oxidation of [Fe-S] clusters and inactivation of the corresponding metalloproteins [41], deamination and oxidative damage of DNA[42], and methionine or lysine deficiency ascribed to interference with succinyl Co-A production [43].

### NO on bone homeostasis

**Fig. 3:**
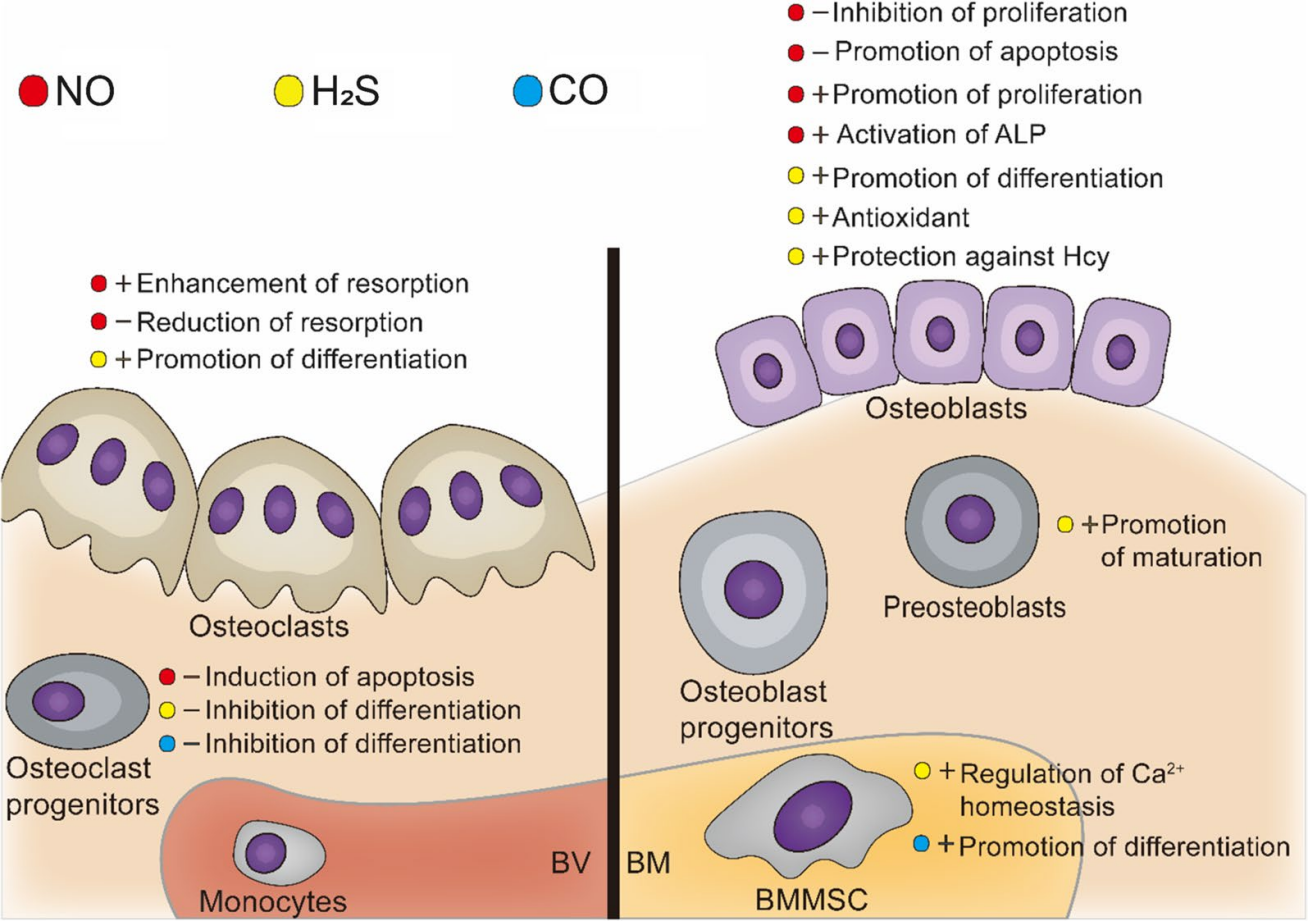
Effect of gaseous signaling molecules on bone homeostasis. ± : promote/inhibit *BV* blood vessel, *BM* bone marrow, *ALP* alkaline phosphatase, *Hcy* homocysteine

NO acts as a biphasic regulator in bone homeostasis. High concentrations of NO released from iNOS within a short period of time inhibit osteoblast proliferation, promote apoptosis [44], and reinforce the resorption of osteoclasts [45]. Low concentrations of NO derived from eNOS intensify alkaline phosphatase activity, drive osteoblast reproduction [46], induce apoptosis of osteoclast progenitors, and curtail the reabsorption of osteoclasts [47]. If bone homeostasis is disrupted, the removal of excess NO or the release of an appropriate amount of NO could probably adjust bone homeostasis to the expected physiological trend to achieve the therapeutic purpose of preventing bone loss or enhancing bone formation (Fig. 3).

### The strategy of local delivery using nanomaterials

Traditional small molecule NO donors are primarily organic nitrates, nitrites, metal-NO complexes, and N-nitrosamines [48]. These NO donors are simple in structure and easily generate NO via enzymatic or nonenzymatic pathways while being deficient in selectivity and therefore rapidly metabolized away. Nevertheless, their selectivity can be boosted by adding target-activated moieties or coupling antibodies for both supplemental functionalities and enhanced intertissued retention of nanoscale macromolecules. This verifies the direct use of nanomaterials for NO delivery as a more desirable and convenient option. The central issue in designing NO nanocarriers is how the NO donor is structured into the material and under what conditions NO is released.

Although the preparation of nanomaterials for the delivery of NO donors is instrumentally essential to the protection of NO donors from being degraded or removed, the use of hollow or porous outer layer nanomaterials for wrapping the NO donor becomes the most common strategy. In addition, the attachment of NO donors onto the surface of nanomaterials by physical or chemical adsorption is another popular strategy [49]. Some of the NO donors themselves can be self-assembled into large nanoparticles, which would provide higher NO release per particle concentration and fewer side effects compared to other nanomaterial frameworks [50].

These NO delivery nanoparticles release NO in diverse ways when accumulating in the destination tissue. Regarding NO donors encapsulated in nanomaterials, the nanomaterials can disintegrate so that the NO donors are released, or NO can be released before the escape of the donors from the nano-framework. NO donors attached by covalent bonding or physical adsorption usually need to be detached to release NO, while self-assembled NO donors can work without depolymerization. All the above release strategies should be dependent on changes in the external environment, either with the difference in the physicochemical environment between pathological conditions and physiological conditions or via artificial activation modes, consisting of changes in the biological environment such as temperature, pH, reactive oxygen levels, oxygen concentration, glutathione (GSH) concentration, and various ion concentrations. Depending on the changes in these indicators, NO can be released selectively and spontaneously. Moreover, ultrasound, heat, near-infrared light (NIR), infrared light, visible light, ultraviolet light (UV), X-rays, or even lasers can be adopted to stimulate the discharge of NO [51, 52].

**Fig. 4:**
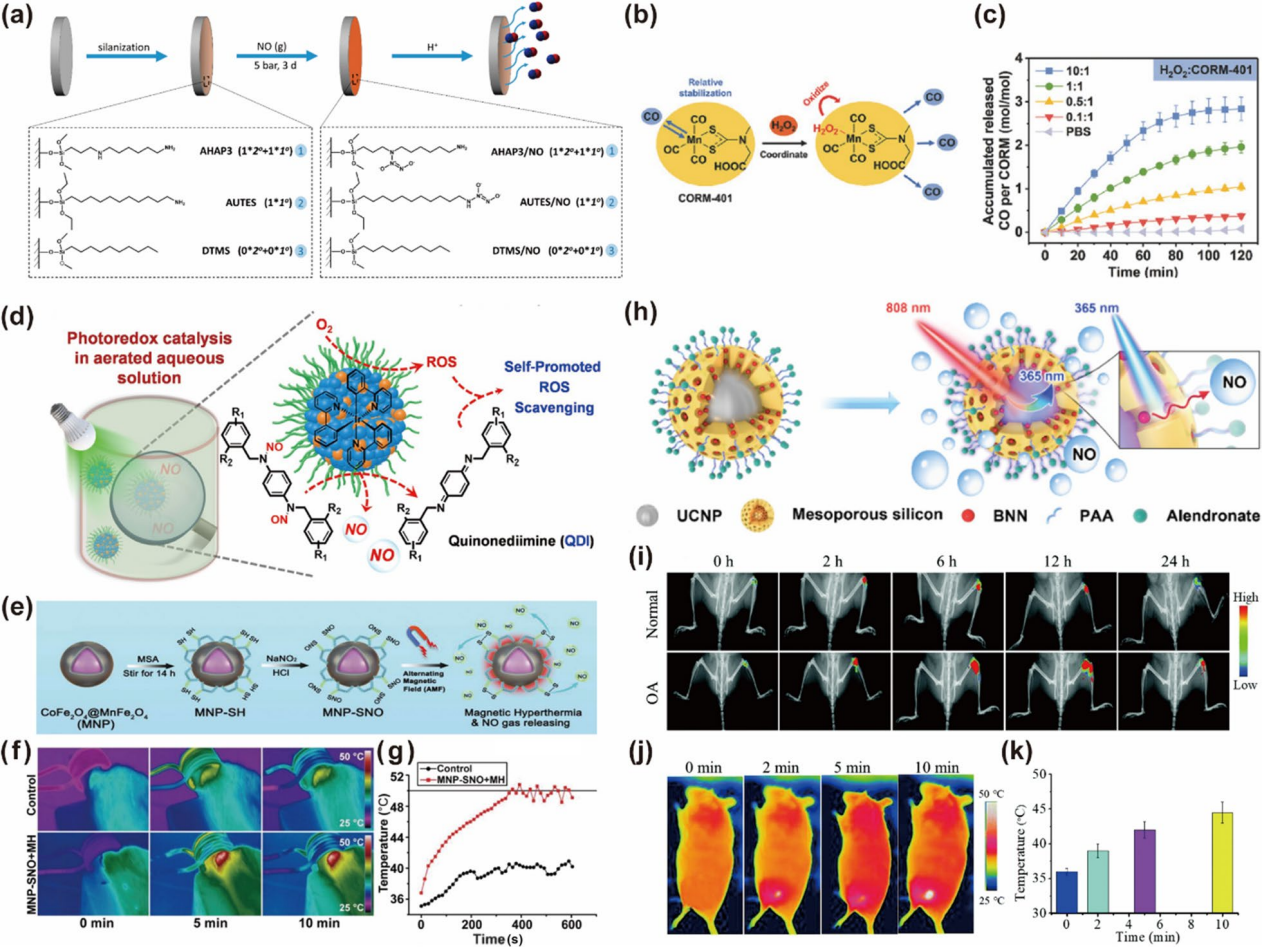
Release strategies for the local delivery of gaseous signal molecules. (**a**) Diazeniumdiolate-functionalized titanium alloy surfaces release NO in an acidic environment. Reproduced with permission from Ref [53]. **b**, **c** CO delivery nanoparticles activated by hydrogen peroxide (H_2_O_2_) to release CO. Reproduced with permission from Ref. [53]. **d** Nano-micelles in an aqueous solution catalyze the release of NO through visible light oxidation. Reproduced with permission from Ref. [55]. **e**–**g** The magneto-thermal effect generated by the alternating magnetic field activates the magnetic nanoparticles to release NO in vivo. Reproduced with permission from Ref. [56]. **h** Conversion of NIR light to UV light in upconversion nanoparticles (UCNPs) activates NO release. Reproduced with permission from Ref [57]. **i**–**k** Photothermally triggered NO nanogenerator releases NO in the joint cavity**.** Reproduced with permission from Ref [58].

With respect to the functions, targeted delivery and controlled release remain the primary functions of nanoplatforms for gaseous signaling molecules in orthopedic diseases. Bacterial colonization changes the environmental pH, and this is no exception in implant-associated infections. Therefore, pH-sensitive gas-releasing surface-modified implants can automatically release NO in the event of periprosthetic infections (Fig. 4a). Cellular oxidative stress in the inflammatory environment of osteoarthritis produces large amounts of H_2_O_2_, which can also be a signal for a biological response (Fig. 4b, c). The spatiotemporal precision and efficiency of gas release are crucial for periprosthetic infections in orthopedics. Near-infrared light can reach deep into the tissue to control gas production. In addition, the application of nano-micelles can significantly curtail the quenching of photocatalytic groups and thus improve efficiency (Fig. 4d). When magnetic nanoparticles are used, the magnetic field controls the depth of tissue released by the gas not only deeper than NIR light, but the accompanying magneto-thermal effect also contributes to antibacterial activity (Fig. 4e–g). Concerning osteoporosis, the drug is expected to act on the surface of a large amount of whole-body bone tissue. The nanoplatform allows for carrying bone-bound alendronate for targeted delivery and upconversion nanoparticles with near-infrared light excitation for controlled release (Fig. 4h). Thermal therapy is commonly employed in the field of osteoarthritis, and the scalability of the nano-drug delivery platform enables us to release gas while being compatible with photothermal effects (Fig. 4i–k).

## Application of NO local delivery nanomaterials

### NO for immune modulation

#### Osteoarthritis

NO is involved in many physiological processes and plays a critical role in the development and progression of illness. Osteoarthritis is a disease characterized by degenerative changes in articular cartilage. Damage to the articular cartilage leads to an initial mild inflammation, followed by the upregulation of iNOS expression by the surrounding chondrocytes, synoviocytes, and macrophages along with the release of a series of inflammatory mediators. Consequently, the relay of a more aggressive cascade of inflammatory responses with the participation of an exceptionally high concentration of NO causes further cartilage degradation [59]. NO not only damages the articular cartilage but also inhibits the synthesis of cartilage proteoglycans by glycosaminoglycans under the stimulation of interleukin-1, hindering cartilage repair [60]. In clinics, patients suffering from either early or progressive osteoarthritis have more iNOS-positive cells in the macrophage, leukocyte, and fibroblast populations of their knee synovium than healthy individuals [61]. These overabundances of NO would considerably contribute to the onset or progression of osteoarthritis. In spite of this, the immune regulation function of NO seems to be able to cure diseases caused by itself.

The role of NO in the immune system has received much attention in research on gaseous signaling molecule delivery nanomaterials since immune modulation can play a crucial role in the treatment of inflammatory diseases. Chen et al. constructed a photothermal-triggered NO-releasing nanoparticle for the treatment of osteoarthritis. They used hemoglobin, a natural photothermal molecule, as the core of the nanoparticles that could be stably loaded with NO at 37 °C. First, these nanoparticles entered the macrophages, and the light energy was converted into heat energy when irradiated by 650 nm near-infrared light, leading the nanoparticles to be unstable and consequently release NO and the inflammatory pathway Notch1 inhibitor-siRNA [58]. This resulted in the downregulation of the proinflammatory Notch1 [62] and P65 [63] pathways and the upregulation of the inflammation-inhibiting AMPA pathway [64]. It demonstrated preferable anti-inflammatory properties, as well as effective attenuation of cartilage erosion due to osteoarthritis in in vivo experiments [58]. However, these nanoparticles were less thermally stable owing to many temperature-sensitive biomaterials and then promoted disintegration at 40 °C. Hence, they are detrimental to the treatment of inflammatory diseases with high localized fever. In addition, these nanoparticles exhibited significant concentration-independent inhibition of macrophage cell viability after irradiation [58], suggesting that this inhibition may be nonspecific and that it may provoke strong side effects.

For osteoarthritis, therapeutic drugs are not the only focus. However, a more accurate staging and grading diagnosis can help clinical decision-making achieve the best treatment outcome. A biodegradable nanoparticle presenting intracellularly generated NO concentration by fluorescence in real-time has been developed [65]. It employs a traditional NO sensing molecule DAF-FM as a reporter, where the total fluorescence intensity is theoretically proportional to the NO concentration. Under normal conditions, pH or other biomolecules, such as dehydroascorbic acid (DHA) and ascorbic acid (AA), can influence the fluorescence intensity of DAF-FM. Moreover, this molecule is limited by its inability to reside inside the cells [66, 67]. Both demonstrate a false signal to the reporter. Encapsulation using poly (lactic-*co*-glycolic acid) (PLGA) helps address these issues. The fluorescence intensity manifested excellent linearity with NO concentration in the model and treatment groups and a significant positive correlation with the severity of the disease. These results demonstrate the prospect of this material for future accurate grading of clinical OA or other NO-involved diseases.

#### Rheumatoid arthritis (NO scavenger)

Rheumatoid arthritis is an autoimmune disease characterized by synovial inflammation and hyperplasia, production of autoantibodies (rheumatoid factor and anti-citrullinated protein antibodies [ACPA]), destruction of cartilage and bone, and systemic cardiovascular, pulmonary, psychological, and skeletal involvement [68]. Excess NO from synoviocytes, macrophages, or fibroblasts was observed in both osteoarthritis and rheumatoid arthritis, making NO likely to induce a high level of TNF-α expression [69–71], which is an essential pathogenic factor in RA. Furthermore, patients with rheumatoid arthritis, as an autoimmune disease, possess significantly higher levels of nitrite in the synovial fluid and serum than patients with osteoarthritis [72]. Decidedly, systemic inflammation attributed to the immune response is more potent than that caused by tissue damage. Initially, the overproduction of NO was considered to transpire in joints before turning into nitrites in the blood, while a study based on clinical samples disproved this perspective. More iNOS was found inside monocytes in the blood of patients with rheumatoid arthritis than in normal subjects, and their activity was higher and significantly correlated with the severity of the disease [73]. In other words, the source of NO in RA articulars may be systemic, enabling local treatment to be close to nonideal. Generally, the terminal autoimmune inflammatory response is tissue destruction due to the massive apoptosis of cells. In the synovial lining and articular cartilage of patients with rheumatoid arthritis, more apoptosis occurs than in healthy individuals and patients with osteoarthritis. The addition of iNOS inhibitors in cultures of explants derived from RA patients reduced this apoptosis, which could be reversed by NO donors [74].

**Fig. 5:**
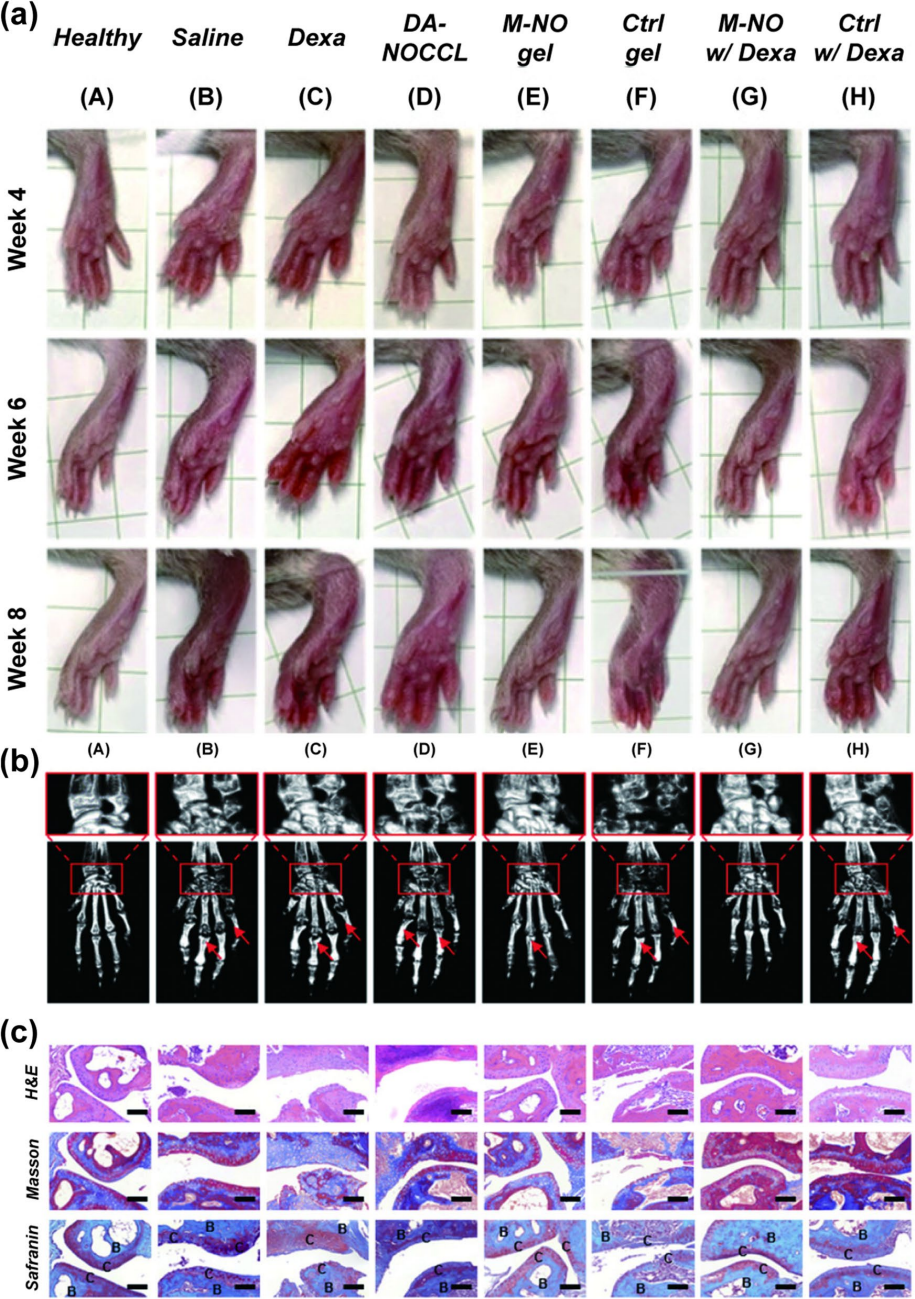
Example of local clearance of NO in rheumatoid arthritis (**a**) Optical images, (**b**) microCT and (**c**) HE staining indicated that M-NO loaded with dexamethasone (M-NO w/Dexa) weakened swelling, bone destruction, and joint surface destruction in the hind paw, respectively. Dexa: dexamethasone, DA-NOCCL: *N*,*N*-(2-amino-1,4-phenylene) dipentyn-4-amide, a dialkyne-functionalized NO-cleavable cross-linker, M-NO gel: polymeric aggregate-embodied hybrid nitric oxide (NO)-scavenging “click” hydrogel, B: bone, C: cartilage. Reproduced with permission from Ref[78].

Kim's team prepared an NO-scavenging hydrogel from acrylamide and an NO cleavage cross-linker, which lessened bone resorption and cartilage destruction better than the commonly used anti-inflammatory drug dexamethasone in a mouse model of adjuvant-induced arthritis [75] (Fig. 5), also known as the RA model. Once NO gas is generated, it immediately and rapidly diffuses with minimal effect by the partial pressure of other gases [76]. Reactive combinations such as the formation of nitrosothiols (RSNO) with thiol groups (RSH) emerge in such large quantities that the diffusion resistance is negligible [77]. Therefore, NO in organisms does not have time to establish concentration gradient-dominated diffusion, even in a phase with uniform temperature. This reflects that the reduction in the local concentration of NO may not be replenished by NO elsewhere on the whole. Additionally, the hydrogel cannot enter the cell or occupy too much volume, leading to swelling of the treatment area due to the increased pressure in the joint cavity. Compared to the active release strategy, this impedes the removal of a certain gas to perform sufficient conditioning, making it more difficult as the gas concentration decreases. Hence, researchers synthesized a novel drug nanocarrier, DA-NOCCL (N,N-(2-amino-1,4-phenylene) dipentyn-4-amide) [78], to alleviate this impediment. When co-injected into the joint with the HA-N_3_ backbone and another cross-linker, PLA-*b*-PEG-N_3_, it can be rapidly gelated in situ by a "click" cycloaddition reaction, contributing to a wider distribution for enhancing the efficiency of NO scavenging. When two or more cross-linkers are used in the self-assembly of small molecules, the resulting hydrogel network generally has an inherent bioactivity that can encapsulate hydrophobic drugs and incorporate bioactive ligands because of the balance of attractive and repulsive forces. Moreover, drug release from these systems can be further guided by the stimulatory transformation of the nanostructure and/or by the cleavage of the environmentally sensitive labile bonds [79]. This gel, for example, can be degraded upon the absorption of NO, inducing DA-NOCCL cleavage and releasing an equal proportion of dexamethasone to intelligently exert a combined anti-inflammatory effect relying on the severity of the disease as symptomized and triggered by the NO concentration [78].

#### Gout

Hyperuricemia, a condition when there is too much uric acid in the body, causes gout. Purines, which can be present in your body and in food, are broken down by the body to produce uric acid. Uric acid crystals, also known as monosodium urate, can accumulate in the body's tissues, fluids, and joints and produce inflammation under excessive amounts. Waldiceu et al. previously identified a ruthenium NO donor [Ru(bpy)_2_(NO)SO_3_] (PF6) that could act as a suppressor of inflammatory pain by activating cGMP/PKG/ATP-sensitive potassium channels and blocking transient receptor potential cation channel subfamily V member 1 (TRPV1) [80]. They subsequently attempted to apply this drug to the joint inflammation of gout. This complex successfully activated the cGMP/PKG/ATP-sensitive potassium channel signaling pathway and targeted monosodium urate crystals (MSU), whose deposition was a causative agent of gout, to curtail its induction of the NALP3 inflammasome, NF-κB, and nociceptive neuron activation [81]. Ruthenium complexes have previously been utilized to kill infectious microorganisms and tumor cells because they can strongly bind nucleic acids and proteins. When used as NO donors, this feature might make the location of NO release more spatially selective [82].

### NO for metabolic regulation

#### Osteoporosis

Osteoporosis is the loss of bone that is not itself symptomatic but rather a systemic impairment of bone mass, strength, and microarchitecture, resulting in an increased propensity for fragile fractures in patients [83]. The mechanism of bone loss is asymmetric bone metabolism, where osteoclast-directed bone resorption is faster than new bone formation by osteoblasts, leading to an imperceptible decrease in the total bone mass. Moreover, the production and behavior of osteoblasts and osteoclasts are highly susceptible to hormones or cytokines, mainly consisting of parathyroid hormone, prostaglandins, insulin, 1.25 (OH)_2_-vitamin D3, epidermal growth factor (EGF), vascular endothelial growth factor (VEGF), and insulin-like growth factor (IGF) [84].

**Fig. 6:**
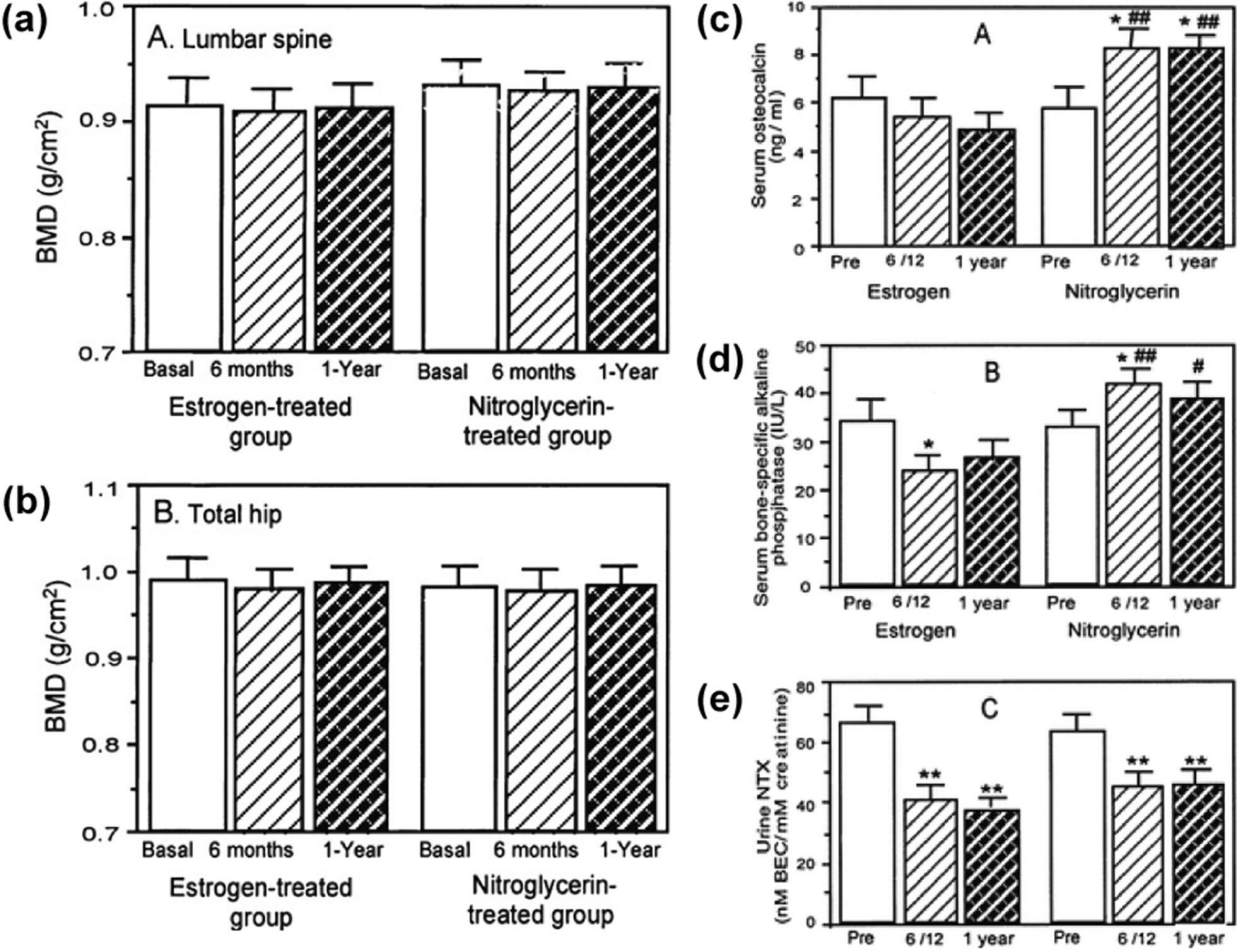
Clinical study of nitroglycerin-releasing NO therapy for bone loss in women after oophorectomy (**a**, **b**) Nitroglycerin therapy is comparable to estrogen replacement therapy in inhibiting BMD decline. Serum osteogenic markers (**c**, **d**) were higher in the nitroglycerin therapy group. Osteoclastic markers (**e**) were comparable to estrogen replacement therapy. Reproduced with permission from Ref [93].

Since the incidence of osteoporosis is significantly higher in menopausal women than in nonmenopausal women and in men of the same age, estrogen deficiency was once the center of osteoporosis research. Estrogen therapy has been effective [85], and several ideas have been proposed regarding the change in bone sensitivity to mechanical stimuli and the regulation of calcium metabolism by estrogen [86, 87]. Nevertheless, the distinct effect of estrogen on cells involved in bone homeostasis has never been clarified. Recent epidemiological and biological evidence suggests that the real cause of bone loss is oxidative stress from aging, which begins before menopause [88]. In research on the antioxidant status of postmenopausal women with osteoporosis, plasma levels of NO markers, especially in erythrocytes [89], were strikingly higher in patients than in healthy individuals [90], in addition to the classical indicators of oxidative stress. For menopausal female athletes, however, NO concentration levels were surprisingly low [91]. Although systemic NO was in excess, releasing NO for OSP therapy in in vivo experiments had a positive effect on bone mineral density (BMD) retention compared to estrogen within a narrow concentration window. Concurrently, clinical trials with oral nitroglycerin remained ineffective [92], while topical skin ointments were effective (Fig. 6) [93]. This may be ascribed to the difference between systemic and local NO.

In addition to the physiological and unavoidable metabolic changes of menopause, type 1 diabetes (T1D), a metabolic disease, can also lead to osteoporosis. Hyperglycemia, insulin deficiency, and insulin resistance in T1D weaken osteoblast-mediated bone formation while boosting osteoclast-mediated bone resorption and fat accumulation in the bone marrow cavity. These negative effects are partly attributed to the inhibition of the PI3K/Akt/NO and NO/cGMP/PKG signaling pathways, which results from the reduced NO bioavailability in bone due to cellular exposure to a high glucose environment [94]. The most etiologically straightforward symptom is inflammation-induced osteoporosis, as commonly seen in autoimmune diseases such as rheumatoid arthritis [95] and ankylosing spondylitis [96]. Inflammatory signaling directly stimulates iNOS expression and NO production. The burst of NO promotes bone resorption, which was observed to be effortlessly reversed by the NOS inhibitor N-methylarginine (L-NMMA) in in vivo experiments [97].

Spatial selective release of NO can be beyond the cellular localization level. Ye et al. targeted sites of high bone turnover using the calcium-chelating molecule bisphosphonate for surface modification of nanoparticles. NO donors in upconversion nanoparticles (UCNPs) were excited by near-infrared light with a characteristic long tissue penetration depth to allow NO release after the deposition of nanoparticles into bone tissue, especially the spine, one of the most frequent sites of osteoporotic fractures [98]. This therapy induced the upregulation of osteogenic gene expression and significantly improved the manifestation of osteoporosis [57]. Bisphosphonates were once used to treat osteoporosis because they bind to bone tissue. They are absorbed by osteoclasts, leading to osteoclast inactivation and apoptosis. However, over-suppression of osteoclasts prevents the removal of old bone and impacts the formation of new bone, which may interfere with bone remodeling or bone repair [99]. Accordingly, this bisphosphonate-reliant bone targeting approach should be adopted with caution.

**Fig. 7:**
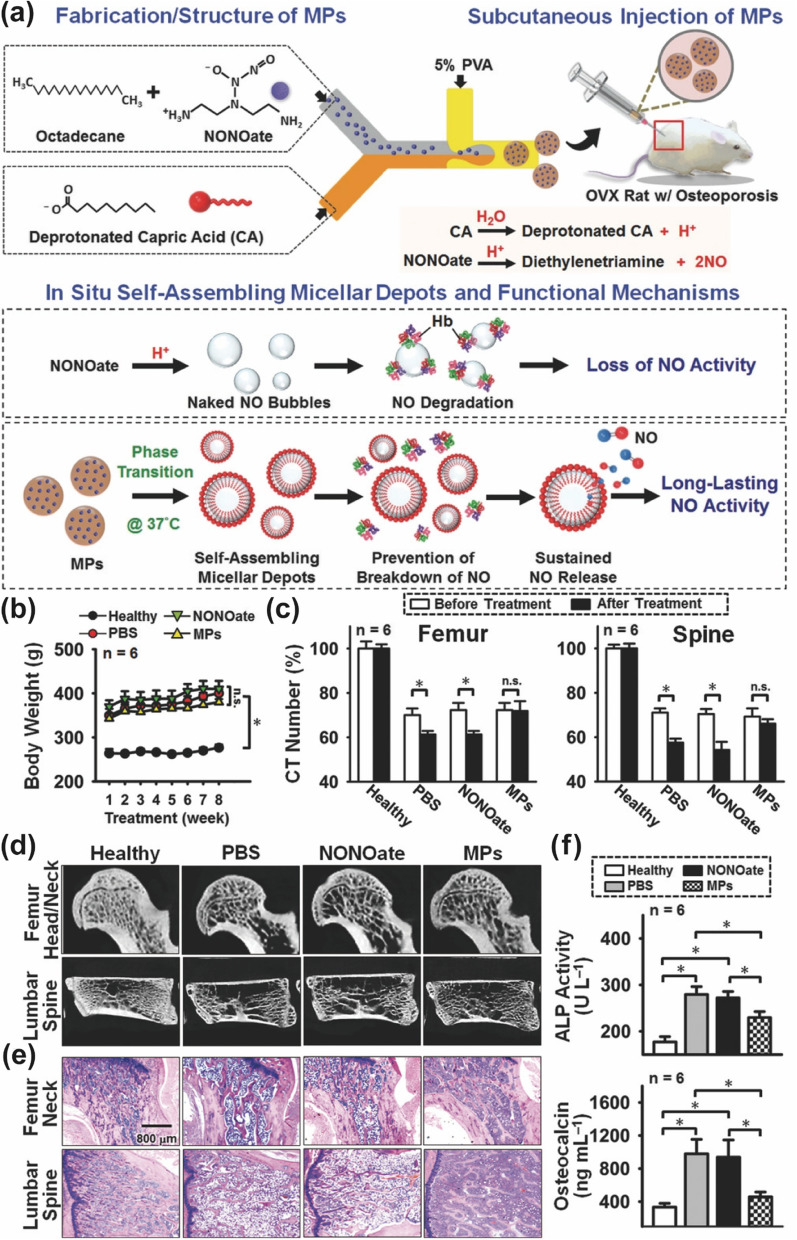
Example of local delivery of NO for the treatment of osteoporosis (**a**) In situ self-assembled microcapsule libraries that actively capture and passively release NO and have persistent activity for the treatment of osteoporosis. **b** The success of ovarian removal mapping is demonstrated. **c** CT numbers, (**d**) reconstructed micro-CT images, and (**e**) H and E staining demonstrate the reversal of bone loss by NO release therapy of various treatments. (**f**) The decline in biomarkers of bone resorption. Reproduced with permission from Ref [101]

Direct delivery of gaseous NO into the organism through physical interactions is challenging for highly reactive molecules such as NO. Molecular NO reacts quickly and strongly with hemoglobin, leaving a few molecules to successfully reach their final destination [100]. Surprisingly, the nanoparticles prepared by Lin et al. with long-chain alkanes and medium-chain fatty acids as the basic framework enhanced the capture of released NO by the embedded donor and underwent a phase transition at 37 °C, causing self-assembly into a nanomicellar depot that competently stored NO bubbles [101] (Fig. 7). Osteoporosis has always been recognized as a disease requiring long-term treatment [102], and the physically protected NO in this study would be released from micelles slowly and persistently, consistent with the understanding of long-term drug use. Long-term NO release therapy has fewer side effects than the widely used long-term bisphosphonates, while the injection method of administration would make compliance much lower in patients who are used to oral administration. Hence, the most convenient approach for administration would be the next potential breakthrough point for NO treatment of osteoporosis.

#### Tendon injuries

The clinical treatment of tendon injuries in recent years has included anti-inflammatory and surgical techniques, but no specific drugs have been widely applied to promote tendon repair [103]. Chen et al. encapsulated NO-releasing MOF inside a highly aligned coaxial polycaprolactone (PCL)/gelatin (Gel) scaffold [104] developed for surgical implantation to replace injured tendons. Similar to osteoporosis, the treatment of tendon injuries is a process requiring chronic repair over a long period. In vitro, NO, which is stable and slowly released for 15 days, promotes tendon repair and maturation through excellent peripheral angiogenesis and vasodilation and thus can rapidly increase blood perfusion within 2 weeks [104]. On the downside, tendon injuries are usually accompanied by inflammation. However, the role played by the immune system in tendon repair, collagen deposition, and the interaction of NO with immune cells remains vague.

As the transmission node of the human musculoskeletal motor system, the physiological and pathological processes of the tendon are significantly influenced by biomechanics, and the tendon bioenvironment is heavily cell-dependent on mechanotransduction [105]. While NO release in the vascular endothelium is closely related to mechanotransduction triggered by shear stress and flow planes [106], the biomechanically relevant role of NO in tendon repair should be further explored.

### NO for antibacterial applications

Implants or prostheses are commonly used in orthopedic surgery to replace or fix damaged bones or joints to help heal or restore functionality. However, implant materials provide shortcuts for microbial invasion, and microbes cause severe clinical problems through mechanisms involving biofilm formation, persistent cell multiplication, immune escape, osteoblast invasion, and antibiotic resistance [107]. Li et al. modified the surface of titanium, a metal commonly used in prosthetic implants, by adding functional groups that can release NO and tested their properties against biofilm formation and promotion of osseointegration [53]. This study was more interested in exploring the chemical release properties of NO loaded on the different carbon atoms of aminosilanes, and in vivo experimental evidence was insufficient. Wang et al. combined NO delivery with magnetothermal therapy to survey the successive release of both NO from its donor on the nanocarrier surface and heat from the magnetic nanoparticles. They discovered that nanoparticles injected around infected implants sharply disrupted mature biofilms when stimulated by alternating magnetic fields. NO also recruits and polarizes macrophages to the M1 state, activating local innate immunity while sterilizing bacteria [56]. The use of magnetic fields to generate heat from magnetic nanoparticles for tumor therapy was proposed as early as the 1950s [108]. However, magnetic fields, which are virtually harmless to the human body, have exhibited an irresistible potential to become a new tool for triggering NO release with advances in materials science over the past century.

### Unspecified problems with NO therapy

#### Difference between exogenous NO and endogenous NO?

When NO is in overabundance, the reduction of NO concentration can be achieved by scavenging NO or inhibiting NOS activity, both of which result in the same essence. While it can be provided by either release from NO donor drugs or increasing NOS activity to produce more endogenous NO when NO is insufficient. Most current artificial NO donors complete their release outside the cell and then allow NO to diffuse into the cell to work, making it different from the behavior of NO released from intracellular NOS. Although cell membranes do not pose a hindrance to small hydrophobic or polar molecules such as NO, changes in the spatial pathways determine the proteins that they react with first, allowing it to differentiate the effects of exogenous and endogenous NO.

It is necessary to have at least comparable concentrations of both sources in the cell to explore whether exogenous and endogenous NO act differently. It is difficult to precisely balance the concentration of NO inside and outside the cell, enabling the same level of a certain effect to study other effects to be a reasonable approach. Researchers focusing on ischemia‒reperfusion (I/R) injury have spiritedly studied this issue [109, 110]. In a study on a rat I/R liver model, only endogenous NO produced oxidative stress on cells by lipid peroxidation and reactive oxygen species formation and reduced leukocyte adhesion and accumulation, while NO donors with an equivalent level of hepatocyte protection had no such effects [111]. This distinction was also found in endothelial cells. Endogenous NO but not exogenous NO can inhibit endothelin-1 production via GC/cGMP-dependent mechanisms [112]. Limited by the paucity of relevant comparative studies, such differences may exist only theoretically, and the positive experimental results obtained may be provoked by the inability to precisely control concentrations. Nevertheless, some studies have instead demonstrated that exogenous and endogenous NO can accomplish the same function [113, 114].

### Scavenge or release NO?

NO plays a subtle role in different diseases, and there are two basic ideas of treatment. First, NO is deficient or in surplus and therefore demands equipoise mechanisms to bring NO concentrations back to normal physiological levels. The second is to exert NO proactive functions for immunomodulation, bactericidal therapy, and regulation of bone homeostasis. The existing understanding of NO in skeletal diseases is presented in Table 2. However, excess NO does not necessarily mean specific removal of NO, and moderate amounts of NO can be applied to immune cells to alleviate inflammation with the same purpose of disease relief. Furthermore, it can still provide therapeutic help through its powerful and diverse biological activity, even if the disease has no relevance to NO.

**Table 2:** Status and effects of NO in orthopedic diseases

Diseases	NO amount	NO-producing cells	Pathological effects	Refs.
Osteoarthritis	Excess	Chondrocytes	Suppressed the biosynthesis of proteoglycan and collagen; Persistently activated NF-κB	[60, 192, 193]
Rheumatoid arthritis	Excess	T cells, Macrophages, Fibroblasts, Synovial cells, Chondrocytes, Osteoclast precursor	Mediated cell apoptosis in the synovium and cartilage Facilitated bone resorption	[31, 72, 74, 194–196]
Osteoporosis	Insufficient	/	Reduced osteocytes response to fluid shear stress Induced osteoblast apoptosis	[89, 91, 197, 198]
Osteonecrosis	Excess iNOS/Insufficient eNOS	Osteocytes/Endothelial cells	Induced osteocytes apoptosis/Led to thrombotic tendency	[199, 200]
Cervical spondylosis	Excess	Nucleus pulposus cell	Unknown	[201, 202]
Ankylosing spondylitis	Controversial	/	/	[203–205]
Gout	Excess iNOS/Insufficient eNOS, nNOS	Chondrocytes/Endothelium, Juxtaglomerular macula densa	Promoted cartilage and bone erosion/Induced hypertension through endothelial dysfunction	[206–209]

## H_2_S

### Physiological effects of H_2_S

Mammalian cells endogenously produce H_2_S mainly by three enzymes: cystathione-γ-lyase (CSE), cystathione-β-synthase (CBS), and 3-mercaptopyruvate sulfotransferase (3-MST) (Fig. 2). Both CBS and CSE use pyridoxal 5′-phosphate (vitamin B6) as a cofactor, while MST requires relay cysteine aminotransferase (CAT) to convert L-cystine, L-cysteine, or homocysteine substrates to H_2_S. Another small fraction of H_2_S is derived from the reduced products of para-sulfur elements when glucose is oxidized in human erythrocytes, and particularly, this is a nonenzymatic reaction [115].

NO acts as an EDRF through a cGMP-related pathway and does not significantly affect the membrane potential. In contrast, H_2_S functions through S-sulfation, which modifies the specific Cys residues in proteins by forming peroxynitrite bonds (− SSH) (Fig. 2) and thus acts as an ATP-dependent potassium ion channel initiator in vascular endothelial cells and smooth muscle cells to trigger hyperpolarization and vasodilation [116]. H_2_S is thus called endothelium-derived hyperpolarization factor (EDHF). Such S-sulfation by H_2_S occurs in large quantities in our body, especially in the liver, and is physiologically indispensable. It was estimated that at least 10% of endogenous GAPDH was sulfated in vivo, which enhanced its activity [117]. Regarding the cardiovascular system, H_2_S can promote angiogenesis [118], inhibit smooth muscle cell proliferation [119], and weaken myocardial ischemia‒reperfusion injury [120, 121].

### H_2_S on immunity, inflammation and bacteria

Although the expression of CBS, CSE and 3-MST is low in all immune cells, H_2_S is still an endogenous anti-inflammatory molecule that plays a role in the differentiation of immune cells and immunomodulation [122]. H_2_S is involved in the initial stages of an inflammatory response. Endogenous H_2_S acts at the leukocyte-endothelial cell interface by inhibiting the expression of adhesion molecules and thus preventing leukocyte adhesion [123]. Then, it is considered an essential endogenous regulator of acute inflammation. In LPS-stimulated macrophages, H_2_S inhibits NO production from iNOS by not only inducing HO-1 expression but also blocking NF-κB activation through the inhibition of IκB degradation and phosphorylation [124]. These findings describe H_2_S as an anti-inflammatory mediator, allowing its interaction with NO to be comprehensively debated later. Moreover, H_2_S-sulfated nuclear transcription factor Y subunit beta (NFYB) promotes ten eleven translocation (Ten) 1 (Tet1) and Tet2 expression for the regulation of T-cell differentiation and maintenance of immune homeostasis [125]. Additionally, H_2_S can promote the polarization of macrophages toward the M2 phenotype under infectious conditions and improve the local immune microenvironment [21], corroborating its role as an inflammation suppressor.

Although the toxicity caused by H_2_S blockage of cellular respiration through binding to the iron of human cell mitochondrial cytochrome enzymes is comparable to that of hydrogen cyanide [126], no research evidence reveals that H_2_S has significant antibacterial activity. The use of H_2_S as an electron donor by sulfur bacteria, a microorganism older than cyanobacteria, transpired long before humans existed. The largest source of H_2_S inside the human body could be our internal gut flora [127], although most of it is excreted and does not enter the circulation while maintaining intestinal mucus integrity and inhibiting pathogenic fragmentation of biofilm and the invasion of pathogenic bacteria [128]. All these results unveil that killing bacteria with H_2_S could be as difficult as killing humans with air components.

### H_2_S on bone homeostasis and orthopedic diseases

Similar to NO, the regulation of bone homeostasis by H_2_S is vital and bidirectional. H_2_S is essential for the maintenance of calcium homeostasis in bone marrow mesenchymal stem cells (BMMSCs), and CBS-deficient mice with low H_2_S levels have lessened bone mass. The S-sulfation of multiple transient receptor potential (TRP) channels by H_2_S initiates subsequent calcium influx and Wnt/β-linked protein signaling to osteogenic differentiation from RUNX2 expression [129]. In the regulation of bone homeostasis, H_2_S production by CSE in osteoblasts enhances the transactivation of RUNX2, promoting osteoblast differentiation and maturation, which is critical in fracture healing [130]. Elevating H_2_S in preosteoclasts downregulates the RANKL/OPG ratio and upregulates NRF2 expression, resulting in the inhibition of preosteoclast differentiation [131]. However, proteomic and transcriptomic studies of H_2_S-producing enzymes yielded opposing conclusions, affirming that CSE contributed to calcium resorption and the expression of osteoclast markers during the early stages of osteoclastogenesis and hence accelerated osteoclast differentiation [132] (Fig. 3). With the wholesomely detailed survey on NO, the bidirectional effect of H_2_S on bone homeostasis is not surprising.

Concerning orthopedic diseases, the role of H_2_S in the various causes of osteoporosis has been extensively investigated. Estrogen deficiency is considered the most important cause of postmenopausal osteoporosis. While recent studies have demonstrated that estrogen can regulate the expression of CBS and CSE in human BMSCs, their inability to produce H_2_S may lead to bone loss [133]. Regarding oxidative stress during this process, H_2_S has also been uncovered to have a protective effect on osteoblasts against H_2_O_2_-caused oxidative damage [134]. H_2_S is a vital factor in protecting osteoblasts from apoptosis due to mitochondrial toxicity induced by homocysteine (Hcy) in hyperhomocysteinemia, a disease caused by vitamin B deficiency that leads to osteoporosis [135]. Additionally, H_2_S inhibits mitochondrial dysfunction and endoplasmic reticulum stress in degenerative diseases and intervertebral disc degeneration (IVDD) [136]. These results imply that physiological concentrations of H_2_S are crucial for the proper functioning of bone tissue, and H_2_S deficiency is probably present in all kinds of degenerative diseases of bone.

### Application of H_2_S local delivery nanomaterials

The current effect of H_2_S-delivered nanomaterials for the treatment of diseases is mainly immunomodulatory or anti-inflammatory. Yu et al. demonstrated a solution to the problem of premature release of the H_2_S donor S-propargylcysteine using polylactic acid (PLA) microspheres and dendritic mesoporous silica nanoparticles, achieved a slow release of H_2_S in vivo for up to 3 days, and observed improved adjuvant-induced arthritis scores, inflammatory factor indices, and imaging performance in rats [137, 138]. S-Propargylcysteine exerts antioxidant effects through the upregulation of the Nrf2-antioxidant response element (ARE) signaling pathway, and its use in treating RA is well established [139]. However, S-propargylcysteine is a CSE-dependent donor and therefore inevitably causes a fluctuation in CSE expression. Thus, systemic administration and local administration should be evaluated to prevent its spread throughout the body since differences exist in CSE expression in different body cells, and this may cause unknown side effects due to elevated H_2_S concentrations elsewhere. Attempts to ensure optimum gas transmitter concentrations to aid diagnosis are not only NO studies. Chen et al. designed a joint fluid H_2_S sensor using a primer–probe and a hybrid chain reaction as the source of the occurring electrochemiluminescence signal, with the strong interaction of the metal ions with H_2_S as the trigger mechanism [140]. Whether such immunomodulators and inflammation inhibitors that mix the intervening effects of immunomodulatory mechanisms can be used to characterize the state of the disease should be explored, regardless of the established link between H_2_S and the clinical symptoms and inflammation indicators in RA patients [141]. Zheng et al. previously developed a pH-responsive H_2_S donor, “JKs” [142], encapsulated in collagen hydrogels, to regulate the microenvironment of intervertebral disc degeneration by releasing H_2_S. H_2_S suppressed nucleus pulposus cell apoptosis, upregulated anabolic protein, downregulated catabolic protein expression, and thus improved IVDD in a rat model by inhibiting the NF-κB signaling pathway and downregulating IL-6 and TNF-α expression [143]. A study involving the systemic administration of a small molecule H_2_S donor for therapy revealed that H_2_S inhibited mechanical pain, grip strength deficits, and depression-like behavior caused by osteoarthritis. The rationale may be to target the central nervous system, where this treatment inhibits microglia activation and maintains high levels of antioxidants in the hippocampus [144]. This demonstrates a potential analgesic-like effect of H_2_S on the nervous system, and it can be used to alleviate a range of orthopedic conditions that cause pain.

**Fig. 8:**
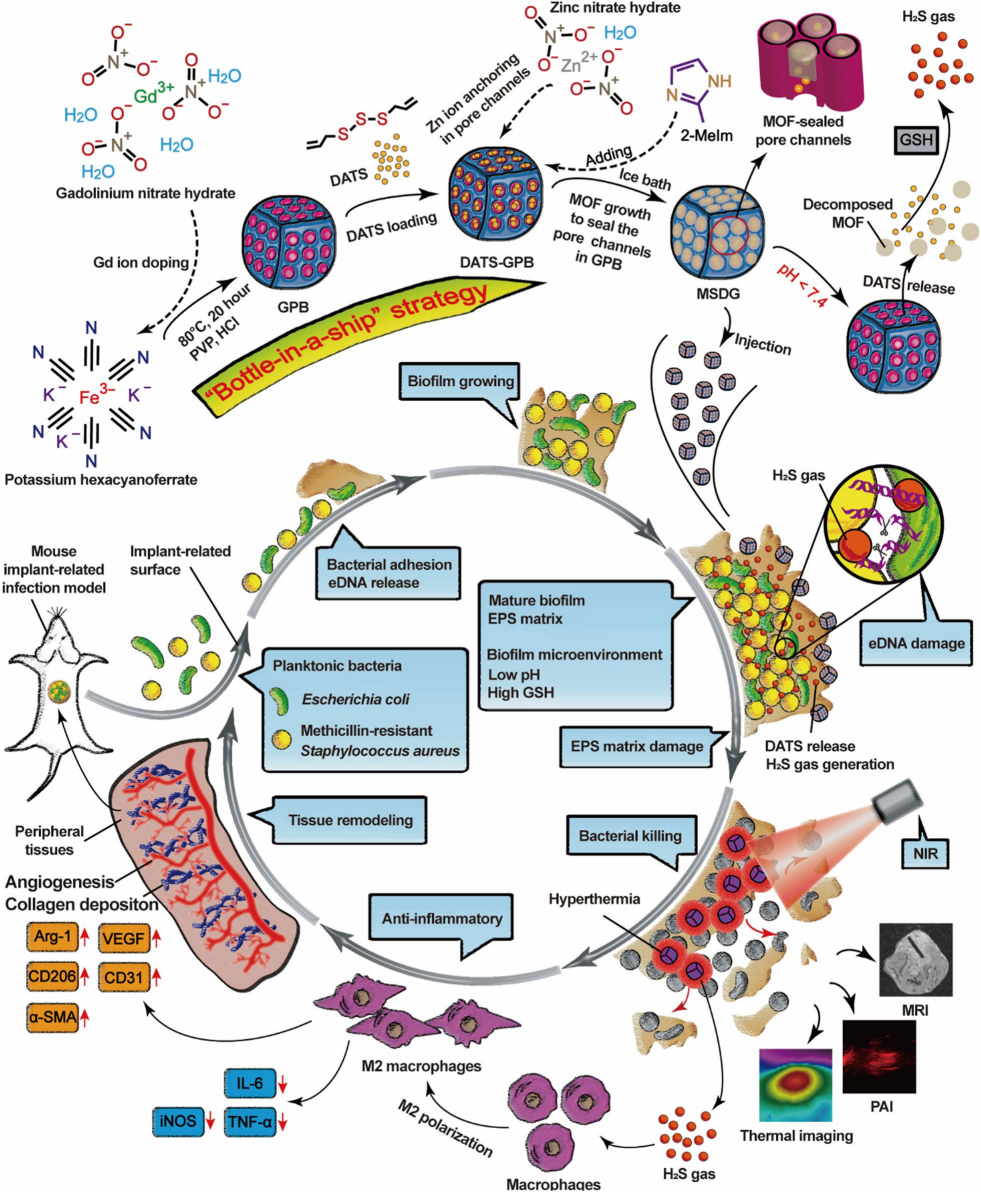
Example of H_2_S used to treat an implant-associated infection The images demonstrate the design, synthesis, and biomedical applications of MSDG. MSDG prepared by the ship-in-bottle strategy reaches the biofilm and initiates the degradation of MOF, which releases DATS. DATS reacts with overexpressed GSH to generate H_2_S gas. With the assistance of NIR laser, H_2_S gas achieves biofilm elimination, immunomodulation of macrophage polarization, inhibition of pro-inflammatory cytokine secretion, and promoted tissue remodeling. GPB: Gd-doped Prussian blue, DATS: diallyl trisulfide, MSDG: metal–organic framework (MOF)-sealed DATS-GPB, PAI: photoacoustic imaging [21]

The antimicrobial activity and potential therapeutic mechanisms of H_2_S have not been extensively studied. However, Su et al. recently developed a strategy of H_2_S-sensitizing thermotherapy and immunomodulation for refractory implant-associated infections, discovering that H_2_S induced eDNA damage in bacterial biofilms and hence split the biofilm and enhanced the effect of thermotherapy on biofilm destruction [21] (Fig. 8). They also employed pH-activated MOF of the microbial membrane environment to trigger the release of the H_2_S donor diallyl trisulfide (DATS), which released H_2_S in response to GSH in the biofilm environment [21]. This dual triggering mechanism greatly improved the effective utilization of H_2_S and weakened the side effects on other tissues.

## CO

### Generation of CO

The first step in the heme degradation pathway is catalyzed by heme oxygenase (HO), the main source of CO in the human body. Heme is catalyzed by heme oxygenase in the presence of oxygen and NADPH to form bilirubin with the release of CO and ferrous ions. Two isozymes, inducible HO-1 and continuous HO-2, are present in the human body. HO-1 is widely distributed throughout the body and is particularly abundant in the spleen, liver, and other tissues that degrade senescent red blood cells [145]. However, it is of little significance to understand the content distribution of HO-1 since the expression of the HMOX1 gene is dramatically upregulated in response to stress generated by various biological, physical, or chemical stimuli. HMOX1 is therefore considered one of the most sensitive and reliable indicators of oxidative stress in cells and one of the most sensitive genes in nature [146].

### Physiological effects of CO

CO molecules easily form coordination bonds with transition metal ions as carbonyl groups. The most abundant transition metal in the human body is iron, and the formation of coordination complexes between CO and ferrous ions is the most typical manifestation of its biological reactivity (Fig. 2). CO strongly binds with many proteins that contain ferrous heme owing to its high affinity, resulting in impaired oxygen-carrying capacity, inhibition of mitochondrial respiration, and platelet activation. In this way, it produces the most widely known toxicity under a large number of deaths and injuries in fires and other accidents yearly [147]. Nevertheless, CO is an essential physiological gaseous signaling molecule that remains indispensable to humans. Similar to NO but with a weaker effect, CO also binds to ferrous ions in sGC and enhances their activity by changing the structure conformation [148]. The resulting cGMP is adopted to regulate vascular tone. Especially under hypoxic conditions, NO can no longer have an effect on cGMP levels in smooth muscle cells when the vascular tone is completely dominated by CO [149].

### Crosstalk among CO, NO, and H_2_S

The catalytic activity of NO binding to sGC is 50 times higher than that of CO binding to sGC [150]. Moreover, the pure binding affinity is 10^8^ times higher [151]. However, both exogenous [152] and endogenous [153] CO have been demonstrated to impair the vasorelaxant effect of the NO-mediated pathway through sGC/cGMP. This could not have been caused by the difference in the concentration of the gas produced at the beginning. One view is that relatively high local concentrations of CO can still be maintained when there is little NO left to diffuse to sGC because CO is much more stable than NO. As a result, the concentration of CO is sufficient to competitively inhibit the binding of NO, while the initiated sGC activity by CO is much lower, allowing for an impactful blocking effect [154]. In addition to competing for the downstream binding sites, CO has regulatory effects on NOS and NO on HO. High concentrations of CO inhibit NOS activity, and crosstalk is achieved through direct binding (heme is also present in NOS [155, 156]). However, physiological concentrations of CO enhance NOS expression, and crosstalk is achieved indirectly through other signaling molecules, such as calcium or phosphoinositide 3-kinase (PI3K) [157]. In parallel, NO can stimulate HO-1 gene transcription and thus increase CO production [158]. Additionally, NO inhibits CSE activity, leading to a reduction in H_2_S production [159]. Conversely, H_2_S can upregulate eNOS and iNOS expression in vascular endothelial cells [160]. In lipopolysaccharide-stimulated macrophages, HO-1 also directly interacts with iNOS via the flavin mononucleotide structural domain, which in turn prevents the binding of iNOS to light chain 3 (LC3) and subsequently endorses lysosomal degradation [161], indirectly contributing to the increased NO concentrations (Fig. 2).

**Fig. 9:**
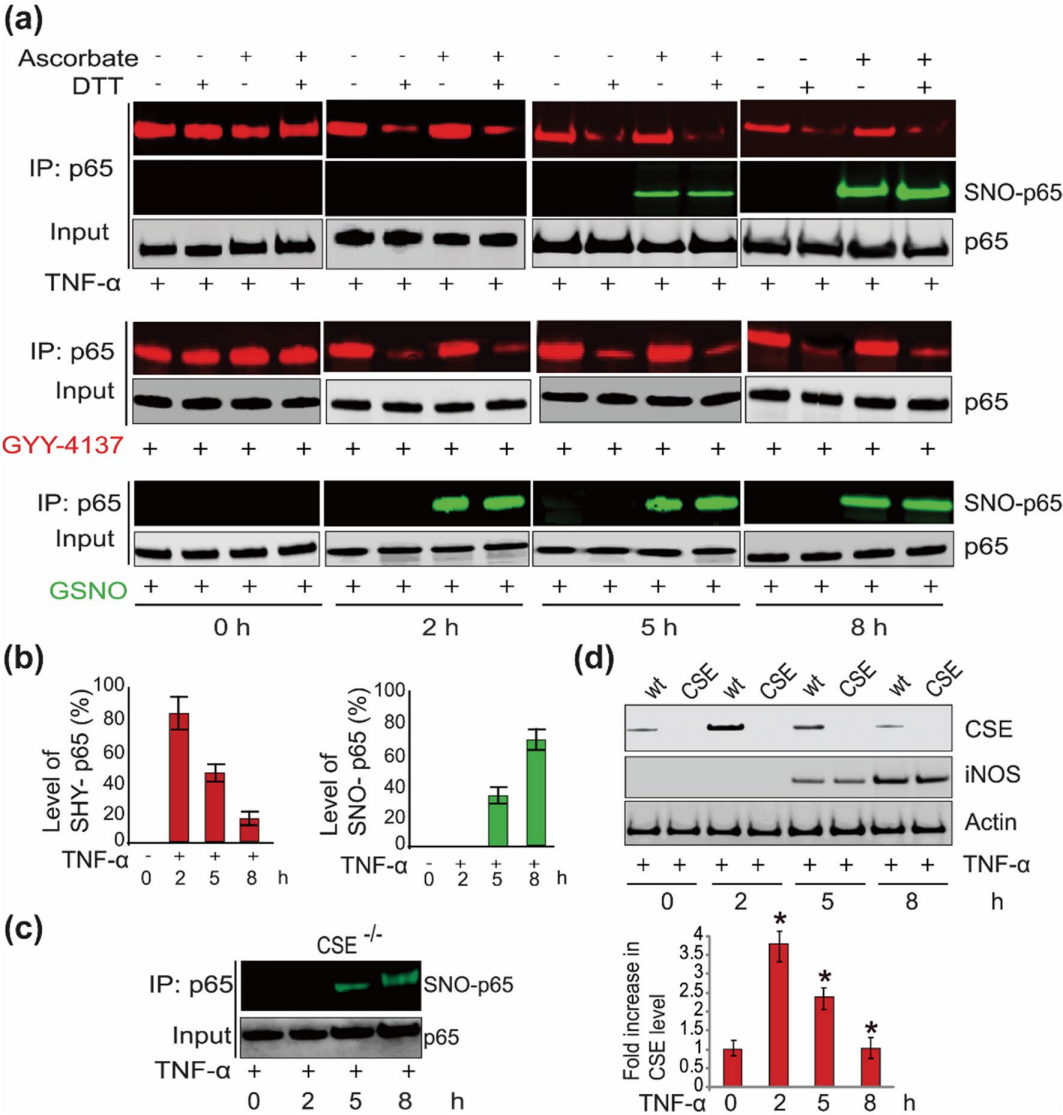
Nitrosylation and sulfhydration of p65 occurred independently in succession (**a**, **b**) Sulfation of p65 in TNF-α-treated macrophages appears early (red fluorescence), and nitrosylation (green fluorescence) appears after its sulfation is reduced. **c** The appearance of nitrosylation is not dependent on the appearance of sulfation. **d** Changes in sulfation and nitrosylation levels are highly consistent with enzyme content. Ascorbic acid and dithiothreitol (DDT) were used to cut off the nitrosylated and sulfated groups on the protein, respectively. GYY-4137: an H_2_S donor, GSNO: S-nitrosoglutathione, NO donor. Reproduced with permission from Ref. (163)

The latest study revealed that the combined application of CO and H_2_S gaseous signaling molecules portrayed a synergistic effect in the treatment of chronic joint pain, producing higher anti-pain and grip strength restoration effects at equal doses [162]. And the p65 subunit of NF-κB was first sulfated by H_2_S produced by CSE and later S-nitrosylated by NO produced by iNOS in TNF-α-induced inflammation and apoptosis. The two processes do not interfere with each other, and the later NO process can also proceed in mice deficient in CSE [163] (Fig. 9). In addition, the combined antibacterial activity of a nano-micelle that released both CO and NO triggered a novel hyperpolarization on the bacterial cell membranes, followed by the initiation of a strong permeabilization effect [20]. Regardless of the presence or absence of crosstalk between these coupled gaseous signaling molecules, most studies in recent years have used only one gaseous signaling molecule. Thus, multigas combinations may still be a pressing issue for future research.

In summary, the three gaseous signaling molecules identified thus far, NO, CO, and H_2_S, have extremely complex crosstalk with each other, involving the effect of the concentration and physiology of either gas. However, only CO, which has a relatively narrow target, deserves greater attention for crosstalk interaction, because NO and H_2_S has so many targets. In other words, the consequence is relatively minor, although identified as a side effect.

### CO on immunity, inflammation, and bacteria

The immunomodulatory role of CO is highlighted in innate immunity, especially in macrophages. In studies of intestinal immunity, exposure to CO or overexpression of HO-1 strengthened the bactericidal activity of mouse macrophages [164]. Mechanistic studies revealed that the endogenous production of CO by macrophages amplified their phagocytosis of bacteria by activation of the NALP3/IL-1β inflammasome [165]. However, exogenous CO negatively regulates the activation of the NALP3 inflammasome by preventing mitochondrial dysfunction [166], suggesting an anti-inflammatory effect of CO. Exogenous CO also binds to the hemoglobin site on oxidase in macrophage mitochondria, causing a rapid and transient burst of ROS. This results in the upregulation of peroxisome proliferator-activated receptor-γ (PPARγ), which exerts anti-inflammatory effects by inhibiting the expression of multiple proinflammatory factors and iNOS [167].

CO can act as a potent anti-inflammatory molecule both in vitro and in vivo. These anti-inflammatory effects mediated by CO are independent of crosstalk with NO or cGMP. CO specifically mediates these anti-inflammatory effects through mitogen-activated protein (MAP) kinases, particularly the MAP kinase kinase (MKK)3/p38 pathway. CO also selectively inhibits the expression of the proinflammatory cytokines TNF-α, IL-1β, and MIP-1β while boosting the production of the anti-inflammatory cytokine IL-10 [168]. Exogenous CO or heme-induced HO-1 can inhibit LPS-induced TNF-α production through the upregulation of the immune response gene 1 (IRG1)/tumor necrosis factor and alpha-inducible protein 3 (TNFAIP3 or A20) pathway to suppress endotoxemia inflammation in mice [169]. This anti-inflammatory effect has been adopted in clinical practice after confirming that CO is fundamentally safe in chronic obstructive pulmonary disease (COPD) patients, with no vital side effects. CO inhalation therapy provoked a reduction in eosinophils in the sputum and improved airway responsiveness to acetylcholine [170]. CO can reduce PTT-induced inflammation in photothermal therapy (PTT) for tumors [171].

Whether CO itself has antimicrobial effects is controversial. The antimicrobial activity of a large number of CO donors may be induced by the transition metal ions contained in the delivery material [172]. Some CO-releasing antimicrobial materials without transition metal ions have been reported, while further investigation is required to confirm the contribution of CO to this antimicrobial role. A transcriptomic study implied that CO, not fully biologically inert, disrupts both genes related to iron acquisition and the metabolism of sulfur amino acids and arginine in bacteria but does not exert a significant antimicrobial effect [173].

### Effects of CO on bone homeostasis and orthopedic diseases

The association of CO with bone homeostasis and orthopedic diseases should be further investigated. CO has been demonstrated to inhibit RANKL-induced osteoclastogenesis (Fig. 3) by suppressing NF-κB-dependent NFATc1 expression. It might be achieved by inhibiting ROS production [174], which was later validated by studies on the inhibition of ovariectomized (OVX) bone loss by CO [162]. CO donors were discovered to have a role in promoting osteogenic differentiation of BMSCs (FBMSCs [3]), while the independent role of CO remains uncertain [175].

Regarding orthopedic diseases, HO-1 was overexpressed in synovial tissue lesions in RA patients in quantities higher than those in OA patients. This indicates that joint tissues may use CO to respond to inflammation, considering that inhibition of HO-1 with siRNA would increase the synthesis of the proinflammatory factors TNFα, IL-6, and IL-8 [176].

### Application of CO local delivery nanomaterials

**Fig. 10:**
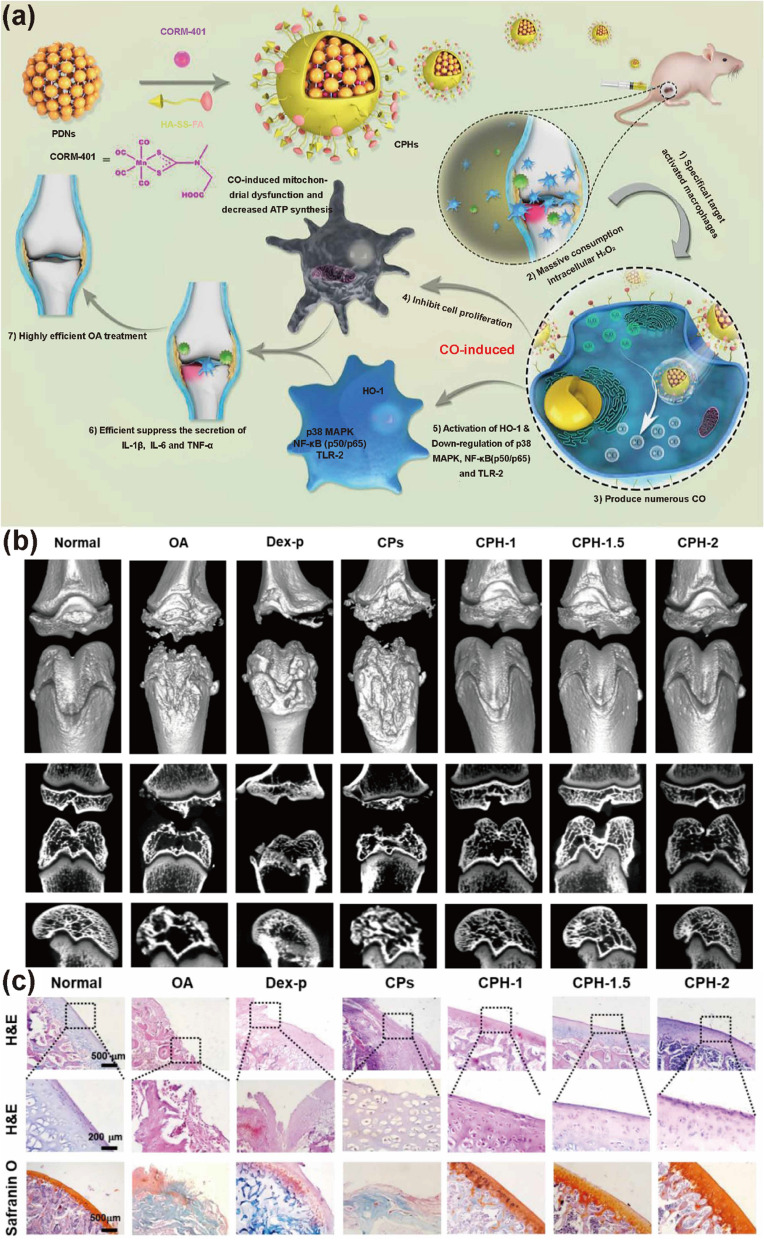
Example of CO-releasing nanoparticles for osteoarthritis (**a**) CO-releasing nanoparticles that can only be activated by consuming hydrogen peroxide. **b** Reconstruction of micro-CT images. **c** H and E and Safranin O staining suggested that CPH reduced cartilage and bone destruction in the knee joint. Reproduced with permission from Ref [179].

Compared with NO and H_2_S, CO is more stable, and its delivery and release are not imminent issues. Therefore, the design strategy of CO nanomaterials mainly focuses on targeting and multifunctionalization. Yang et al. developed a nanomaterial using HA-modified FA-wrapped CO donors by exploiting the surface expression of hyaluronic acid (HA) [177] and folate (FA) [178] receptors on activated macrophages (Fig. 10). The loaded NO donor was H_2_O_2_-responsive, which lessened the excess ROS in OA. In addition to inhibiting inflammatory factors and suppressing inflammatory pathways such as p38 MAPK and NF-kB, this CO-releasing drug unexpectedly activated the expression of HO-1, contributing to a greater anti-inflammatory effect [179]. Yuan et al. have addressed the difficulty of balancing the high-temperature-caused tissue destruction from the single therapeutic use of PTT by augmenting and enhancing the combined therapy of PTT with CO-releasing nanoparticles on the surface of implant-associated infections for antimicrobial treatment. After the pathogens are eliminated, CO continues to exert a powerful anti-inflammatory effect by inhibiting M1 inflammatory factor expression and promoting M1 macrophage polarization to M2 to rapidly adjust the tissue’s internal environment to a repair and osteogenesis-promoting mode. The conditions for CO release are relatively vague, although this gentler therapy has achieved remarkable results in in vivo experiments [180]. Postsurgical wound infections are likewise quite problematic and likely to cause systemic diseases, whereby the bacterial pathogen *Staphylococcus aureus* is the leading cause of skin and soft tissue infections in humans [181]. The photooxidation-activated CO-releasing nanomaterial developed by Cheng et al. can eradicate MRSA infections and accelerate their wound healing after the nano-micelles are selectively absorbed, particularly by *Staphylococcus aureus*, before the intracellular release of CO upon red light irradiation [182]. MRSA is rarely found in the normal human microbial community, while E. coli, which cannot absorb these nano-micelles, usually thrives. Therefore, this material may eradicate invasive bacteria by precisely eliminating pathogenic bacteria to restore the physiological microbial environment, providing a new paradigm for antimicrobial drugs.

## Conclusions

Considering the current frequent use of intra-articular and intramuscular injections in orthopedics, future nanomaterial-mediated gaseous signaling molecular therapies for drug delivery will continue the local injections used in existing studies. Thus, first-pass effects and targeted delivery at the organ level can be almost disregarded. Given the wide range of gaseous signaling molecule targets, delivery to specific populations of cells or even to the organelles or proteins within would be a more distant target. The concentration-dependent variation in the action of gaseous signaling molecules indicates that precise modulation of local concentrations in the controlled release is equally critical. The transient nature of the gaseous signaling molecules at work equips the diffusion of donor molecules with a significant impact on local concentrations, although the desired range of optimal efficacy can be obtained experimentally. Therefore, it may be more vital for researchers to achieve a quantitatively homogeneous and ideal distribution of donor molecules at the lesion site before releasing the gas, which even involves anatomical issues at the macroscopic level. Modern orthopedics is a medicine that makes extensive use of implants. Furthermore, diseases in joint surgery generally start with inflammation and implantation of joint prostheses when symptoms are severe, leading to postoperative infection problems. Gaseous signaling molecules that span immunomodulation, metabolic regulation of bone growth, and antimicrobial activity can simultaneously benefit patients at all three levels. Therefore, the delivery of gaseous signaling molecules to implants is of particular interest.

Currently, no nanomedicines that release or scavenge gaseous signaling molecules have been listed or are in clinical trials. However, small molecule drugs for the same function for orthopedic diseases are paving the way. As demonstrated previously, nitroglycerin ointment for the prevention of postmenopausal osteoporosis has had good results in clinical trials. OrthoDerm, a transdermal nitroglycerin patch for the treatment of chronic tendonitis used in patients with tennis elbow, appears to be stalled in Phase II clinical trials. In terms of scavenging therapies, GW274150, sponsored by GlaxoSmithKline, for RA, and SD-6010, sponsored by Pfizer, for osteoarthritis of the knee, have both completed phase II clinical trials.

In conclusion, more localized gaseous signaling molecule delivery therapies will emerge with a deepening understanding of their physiological role. The maturation and clinical introduction of these diverse therapies will be facilitated by developments in nanodrug delivery technology. Recent developments suggest that its future is unceasingly bright, despite the ongoing debate regarding this therapy’s local concentration, biological impact, safety, and antimicrobial activity. The use of nanomedicines in bones and joints is much less risky and easier to control than in other organs because orthopedic diseases are closely linked to bone homeostasis, immunity, microbiology, and metabolism. Thus, the treatment of orthopedic diseases would be one of the most feasible, effective, and pioneering areas for the future application of local gaseous signaling molecule delivery therapies.

## Data Availability

Data sharing is not applicable to this article, as no datasets were generated or analyzed in the current study.

## Abbreviations

NO: Nitric oxide
H_2_S: Hydrogen sulfide
CO: Carbon monoxide
OA: Osteoarthritis
RA: Rheumatoid arthritis
OSP: Osteoporosis
IVDD: Intervertebral disc degeneration
NOS: Nitric oxide synthase
eNOS: Endothelial NOS
nNOS: Neuronal NOS
iNOS: Inducible NOS
mtNOS: Mitochondrial NOS
HO: Heme oxygenase
CBS: Cystathionine β-synthase
CSE: Cystathionine γ-lyase
3-MST: Mercaptopyruvate sulfurtransferase
sGC: Soluble guanylyl cyclase
EDRF: Endothelium-derived relaxing factor
cGMP: Cyclic guanosine monophosphate
OPC: Osteoclast precursor
BM: Bone marrow
TIMP: Tissue inhibitor of metalloproteinase
MMP: Matrix metalloproteinase
NF-κB: Nuclear factor kappa-light-chain-enhancer of activated B cells
AP-1: Activator protein 1
JAK: Janus kinase
STAT: Signal transducer and activator of transcription
ALP: Alkaline phosphatase
ACPA: Anti-citrullinated protein antibodies
TNF-α: Tumor necrosis factor α
ROS: Reactive oxygen species
EGF: Epidermal growth factor
IGF: Insulin-like growth factor
VEGF: Vascular endothelial growth factor
BMD: Bone mineral density
T1D: Type 1 diabetes
PI3K: Phosphoinositide 3-kinase
Akt: Protein kinase B
L-NMMA: *N*-Methylarginine
I/R: Ischemia‒reperfusion
GSH: Glutathione
siRNA: Small interfering RNA
Notch1: Neurogenic locus notch homolog protein 1
AMPA: α-Amino-3-hydroxy-5-methyl-4-isoxazolepropionic acid
DAF-FM: Diaminofluorescein FM
DHA: Dehydroascorbic acid
AA: Ascorbic acid
PLGA: Poly (lactic-co-glycolic acid)
MicroCT: Micro computed tomography
HE: Hematoxylin–eosin
TRPV1: Transient receptor potential vanilloid subfamily 1
ATP: Adenosine triphosphate
MSU: Monosodium urate
NALP3: NLR family pyrin domain containing 3
UCNPs: Upconversion nanoparticles
MOF: Metal organic framework
PCL: Polycaprolactone
Gel: Gelatin
CAT: Cysteine aminotransferase
EDHF: Endothelium-derived hyperpolarizing factor
GAPDH: Glyceraldehyde-3-phosphate dehydrogenase
BMMSCs: Bone marrow mesenchymal stem cells
TRP: Transient receptor potential
RUNX2: Runt-related transcription factor 2
RANKL: Receptor activator of nuclear factor-kappa B ligand
OPG: Osteoprotegerin
NRF2: Nuclear erythroid 2-related factor 2
Hcy: Homocysteine
NFYB: Nuclear transcription factor Y subunit beta
Tet1: Ten-eleven translocation 1
Tet2: Ten-eleven translocation 2
DATS: Diallyl trisulfide
HMOX1: Heme oxygenase 1
LC3: Light chain 3
PPARγ: Peroxisome proliferator-activated receptor-γ
MAP: Mitogen-activated protein
MKK: Mitogen-activated protein kinase kinases
MIP-1β: Macrophage inflammatory protein 1β
IRG1: Immune response gene 1
TNFAIP3: Tumor necrosis factor alpha induced protein 3
COPD: Chronic obstructive pulmonary disease
PTT: Photothermal therapy
NFATc1: Nuclear factor of activated T cells 1
OVX: Ovariectomized
BMSC: Bone mesenchymal stem cells
HA: Hyaluronic acid
FA: Folate acid
MRSA: Methicillin-resistant *Staphylococcus* aureus
